# Supplementary material for: High Substitution Synthesis of Carboxymethyl Chitosan for Properties Improvement of Carboxymethyl Chitosan Films Depending on Particle Sizes
Source: Molecules. 2021 Oct 3;26(19):6013. doi: 10.3390/molecules26196013 (PMC8512063; doi:10.3390/molecules26196013)
Supplement: Supplementary file 1 [file molecules-26-06013-s001.zip › molecules-1315315-supplementary.pdf]

Supplementary Materials

High Substitution Synthesis of Carboxymethyl Chitosan for Properties Improvement of Carboxymethyl Chitosan Films Depending on Particle Sizes

Sarinthip Thanakkasaranee, Kittisak Jantanasakulwong , Yuthana Phimolsiripol, Noppol Leksawasdi, Phisit Seesuriyachan, Thanongsak Chaiyaso, Pensak Jantrawut, Warintorn Ruksiriwanich, Sarana Rose Sommano, Winita Punyodom, Alissara Reungsang, Thi Minh Phuong Ngo, Parichat Thipchai, Wirongrong Tongdeesoontorn and Pornchai Rachtanapun*

Table S1 The average particle size of chitosan and CMCh powders analyzed by SEM analysis.

Mesh size	Chitosan		CMCh	
	Width(μm)	Length (μm)	Width (μm)	Length (μm)
75 mic	58.2-73.4	125.2-132.0	87.7-94.4	113.0-132.0
	(Avg. 67.7)	(Avg. 128.8)	(Avg. 91.0)	(Avg. 126.0)
125 mic	92.8-115.7	162.2-221.4	117.4-121.4	140.3-267.4
	(Avg. 103.3)	(Avg.186.0)	(Avg. 119.6)	(Avg. 196.9)
250 mic	167.1-233.2	356.2-505.8	212.9-275.0	329.3-517.8
	(Avg.210.2)	(Avg.446.5)	(Avg.276.4)	(Avg.420.9)
425 mic	354.1-467.0	639.1-696.4	448.7-484.6	563.8-629.0
	(Avg.426.8)	(Avg.623.5)	(Avg.461.0)	(Avg.599.1)
850 mic	326.4-602.2	629.3-886.4	340.45-714.3	731.4-1073.7
	(Avg.433.9)	(Avg.751.2)	(Avg.583.2)	(Avg.819.6)

Avg.: average size

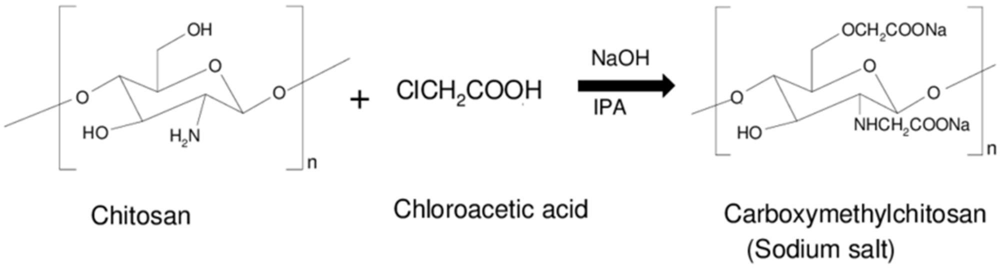

Figure S1. Schematic of carboxymethyl chitosan reaction [1].

[1] Bedekar, A.N., Pise, A.C., Thatte, C.S. and Rathnam, M.V., 2010. Study on optimization of carboxymethylation of chitosan obtained from Squilla chitin. Asian Journal of Chemistry, 22(10), p.7675.
